# Characterization of an engineered mucus microenvironment for in vitro modeling of host–microbe interactions

**DOI:** 10.1038/s41598-022-09198-6

**Published:** 2022-04-01

**Authors:** Andy J. Huang, Courtney L. O’Brien, Nicholas Dawe, Anas Tahir, Alison J. Scott, Brendan M. Leung

**Affiliations:** 1 School of Biomedical Engineering, Faculties of Medicine and Engineering, Dalhousie University, Halifax, NS B3H 4R2 Canada; 2 Department of Applied Oral Sciences, Faculty of Dentistry, Dalhousie University, Halifax, NS B3H 4R2 Canada; 3 Department of Pathology, Faculty of Medicine, Dalhousie University, Halifax, NS B3H 4R2 Canada; 4 Department of Process Engineering and Applied Science, Faculty of Engineering, Dalhousie University, Halifax, NS B3H 4R2 Canada

**Keywords:** Microbiology, Biomaterials

## Abstract

The human mucus layer plays a vital role in maintaining health by providing a physical barrier to pathogens. This biological hydrogel also provides the microenvironment for commensal bacteria. Common models used to study host–microbe interactions include gnotobiotic animals or mammalian–microbial co-culture platforms. Many of the current in vitro models lack a sufficient mucus layer to host these interactions. In this study, we engineered a mucus-like hydrogel Consisting of a mixed alginate-mucin (ALG-MUC) hydrogel network by using low concentration calcium chloride (CaCl_2_) as crosslinker. We demonstrated that the incorporation of ALG-MUC hydrogels into an aqueous two-phase system (ATPS) co-culture platform can support the growth of a mammalian monolayer and pathogenic bacteria. The ALG-MUC hydrogels displayed selective diffusivity against macromolecules and stability with ATPS microbial patterning. Additionally, we showed that the presence of mucin within hydrogels contributed to an increase in antimicrobial resistance in ATPS patterned microbial colonies. By using common laboratory chemicals to generate a mammalian–microbial co-culture system containing a representative mucus microenvironment, this model can be readily adopted by typical life science laboratories to study host–microbe interaction and drug discovery.

## Introduction

The human mucus layer is known to play a crucial role in maintaining health and providing protection for the underlying epithelial cells. In areas such as the respiratory and gastrointestinal (GI) tract, the mucus hydrogel lining provides a microenvironment inhabited by both commensal and pathogenic bacteria^1,2^. Mucins are the backbone of the mucus hydrogel network, which are highly glycosylated peptides that are interconnected through both covalent and non-covalent interactions^3^. Under healthy conditions, the mucus microenvironment is highly regulated and acts as a selectively diffusive barrier containing secreted immunoglobulins and antimicrobial peptides to help maintain the microbiota load^4,5^. However, mucus dysregulation can lead to alterations in mucus composition and serious health complications^6^. For example, in cystic fibrosis (CF), dehydration and thickening of lung mucus caused by mutations in the *cftr* gene^7,8^ enables opportunistic pathogen *Pseudomonas aeruginosa* to enter a mucoid state, leading to increased antibiotic resistance and chronic infection^9,10^.

To study the interactions between host and pathogen, it is important to provide the proper mucus microenvironment for these interactions to take place. In recent years, there has been interest in modeling these interactions in vitro with mammalian–microbial co-culturing techniques, including transwell co-culture and microfluidic platforms^11–13^ which supports the differentiation of mammalian cells to produce their own mucus layer. However, they are often limited by the low volume of mucus that is produced and the long culture periods to produce them^11,14,15^. Recently, our group has applied the aqueous two-phase system (ATPS) co-culture platform to microbial colony patterning and compartmentalization by utilizing the interfacial tension between two immiscible polymer solutions containing polyethylene glycol (PEG) and dextran (DEX) to contain bacterial growth^13,16^. While ATPS can support direct microbial colony growth over a mammalian monolayer, the PEG component can pose cytotoxicity to mammalian cells in a dose and time dependent manner^17,18^. In addition, this direct co-culture configuration does not account for the spatial distribution of microbes over and within a physiologically relevant mucus layer, thus an engineered hydrogel mucus mimic may serve to better represent host–microbe interactions.

With the aim of providing a suitable mucus microenvironment for in vitro host–pathogen interaction, we have designed and fabricated a mucus-like hydrogel that could be overlaid onto a mammalian monolayer within a PEG-DEX ATPS co-culture platform. Additionally, the incorporation of a mucus-like hydrogel layer into the PEG-DEX ATPS platform can also act as a selectively diffusive barrier to mitigate PEG-cytotoxicity. The hydrogel network that was chosen to fabricate this mucus-like hydrogel layer was alginate due to its ease of use, cytocompatibility and non-cell adherent properties^19^, where mucin was incorporated to mimic the biochemical properties of natural mucus. The compatibility of the resulting mixed alginate-mucin (ALG-MUC) hydrogels with a PEG-DEX ATPS was assessed based on its cytocompatibility and ATPS formation. Furthermore, physical characteristics, such as viscoelasticity and bulk molecular diffusivity, of the ALG-MUC hydrogels were characterized. Lastly, the utility of the ALG-MUC hydrogels was demonstrated by supporting the simultaneous growth of bacterial colonies and mammalian cells using ATPS co-culture. We expected that this combination of hydrogel and polymer-based liquid microbial scaffold would enable the recapitulation of spatial–temporal complexities of the human microbiome niche.

## Results

### The presence of mucin and the ATPS formulation used determines where bacteria preferentially grow

**Figure 1:**
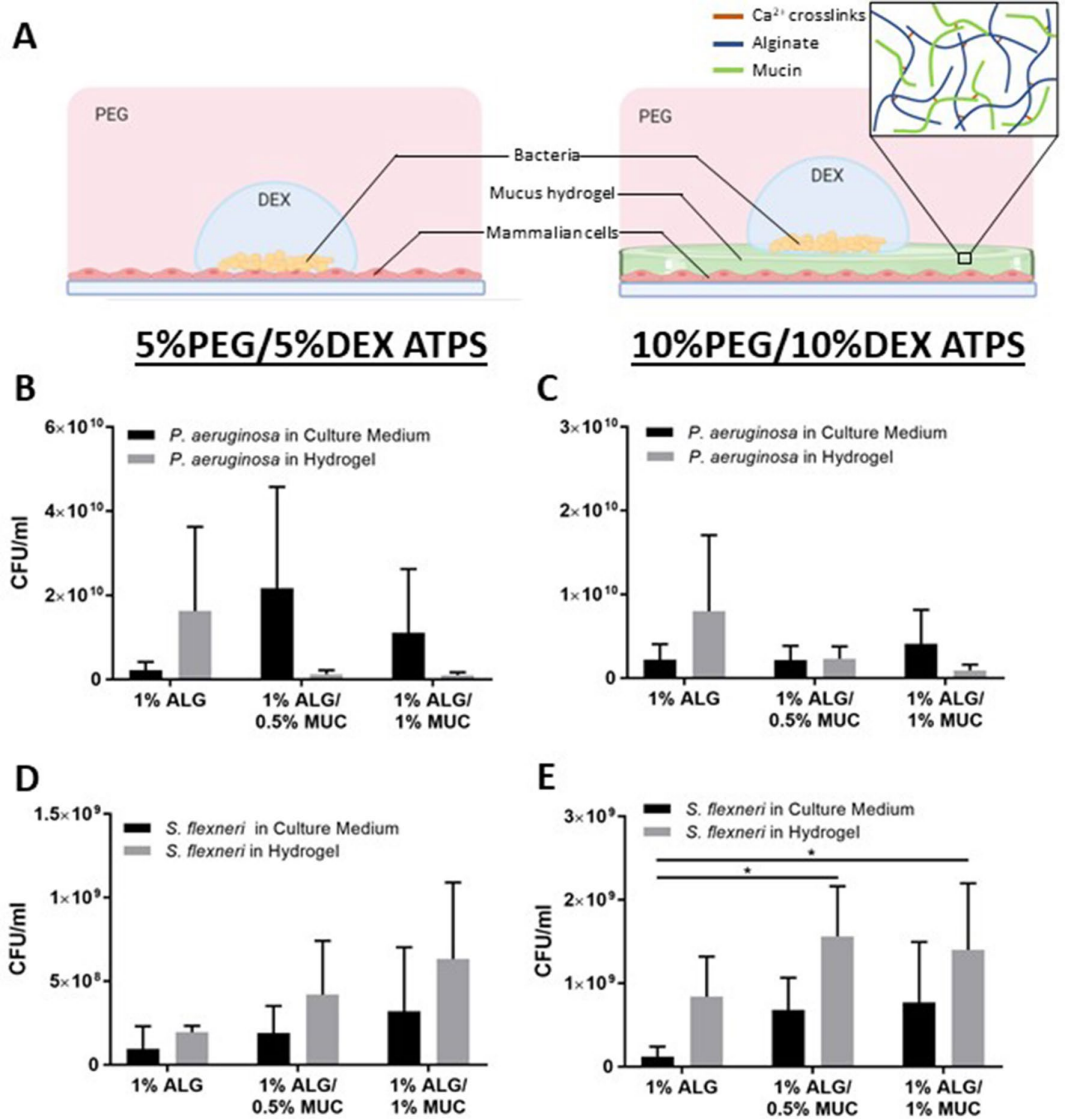
*P. aeruginosa* and *S. flexneri* abundance (CFU/ml) within the culture medium or hydrogel layer after 12 h of co-culture with 16-HBE and Caco-2, respectively. (**A**) Illustration of a PEG-DEX ATPS mammalian–microbial co-culture without (left) and with (right) an engineered mucus hydrogel layer, illustrating all potential crosslinking interactions within the mixed ALG-MUC hydrogel network. CFU/ml values of *P. aeruginosa* grown in either a 5%PEG/5%DEX ATPS (**B**) or 10%PEG/10%DEX ATPS (**C**), and *S. flexneri* in a 5%PEG/5%DEX ATPS (**D**) or 10%PEG/10%DEX ATPS (**E**), where *indicates *p* < 0.05 (n = 4).

In this study, we established a mammalian–microbial co-culture using a PEG-DEX ATPS containing an engineered mucus-like hydrogel, where mammalian cells were overlaid with the hydrogel layer followed by bacterial deposition contained within the DEX-rich phase, as illustrated in Fig. 1A. Two sets of mammalian–microbial co-cultures were carried out: 16-HBE cells with *P. aeruginosa* and Caco-2 cells with *Shigella flexneri* to provide scenarios of host–pathogen interaction within the airway and the GI tract, respectively. We were interested in understanding the bacterial distribution and the behavior of flagellated (*P. aeruginosa*) and non-flagellated (*S. flexneri*) bacteria within the co-culture system; therefore, we assessed the bacterial abundance within the different hydrogel layers compared to the culture medium (PEG-phase and DEX-phase). Although not statistically significant, in comparison to the culture medium, *P. aeruginosa* were found to be more abundant in the 1% ALG hydrogel layer and lower in abundance in the ALG-MUC hydrogels (1% ALG/0.5%MUC and 1% ALG/1% MUC) compared to the abundance in the respective culture medium components, when using a 5% PEG/5% DEX ATPS (Fig. 1B). When co-cultured using a 10% PEG/10% DEX ATPS, the abundance trend held true when *P. aeruginosa* were grown on a 1% ALG and 1% ALG/1% MUC hydrogel but not on the 1% ALG/0.5% MUC hydrogel, where the abundance of bacteria in the culture medium to the hydrogel layer was roughly even (Fig. 1C).

For *S. flexneri*, on the other hand, bacterial abundance was observed to be consistently higher within the hydrogel layers for all hydrogel formulations compared to the culture medium (Fig. 1D,E). Additionally, the bacterial abundance was found to be increasing with increasing mucin concentration within the hydrogels, where the differences in bacterial abundance were found to be more pronounced when using a 10% PEG/10% DEX ATPS compared to a 5% PEG/5% DEX ATPS (Fig. 1D,E).

### Supplemented antibiotic at MIC reduces the abundance of bacteria within the culture medium while maintaining microbial growth within hydrogel layer

The mucus layer is known to be a selectively diffusive barrier, which impacts antibiotic efficacy and provides a microenvironment for bacterial proliferation^20,21^. Therefore, we were interested in assessing the bacterial distribution within our co-culture system after exposure to ciprofloxacin at minimum inhibitory concentrations (MIC), which was determined to be 0.125 μg/ml in suspension cultures for both *P. aeruginosa* and *S. flexneri*.

**Figure 2:**
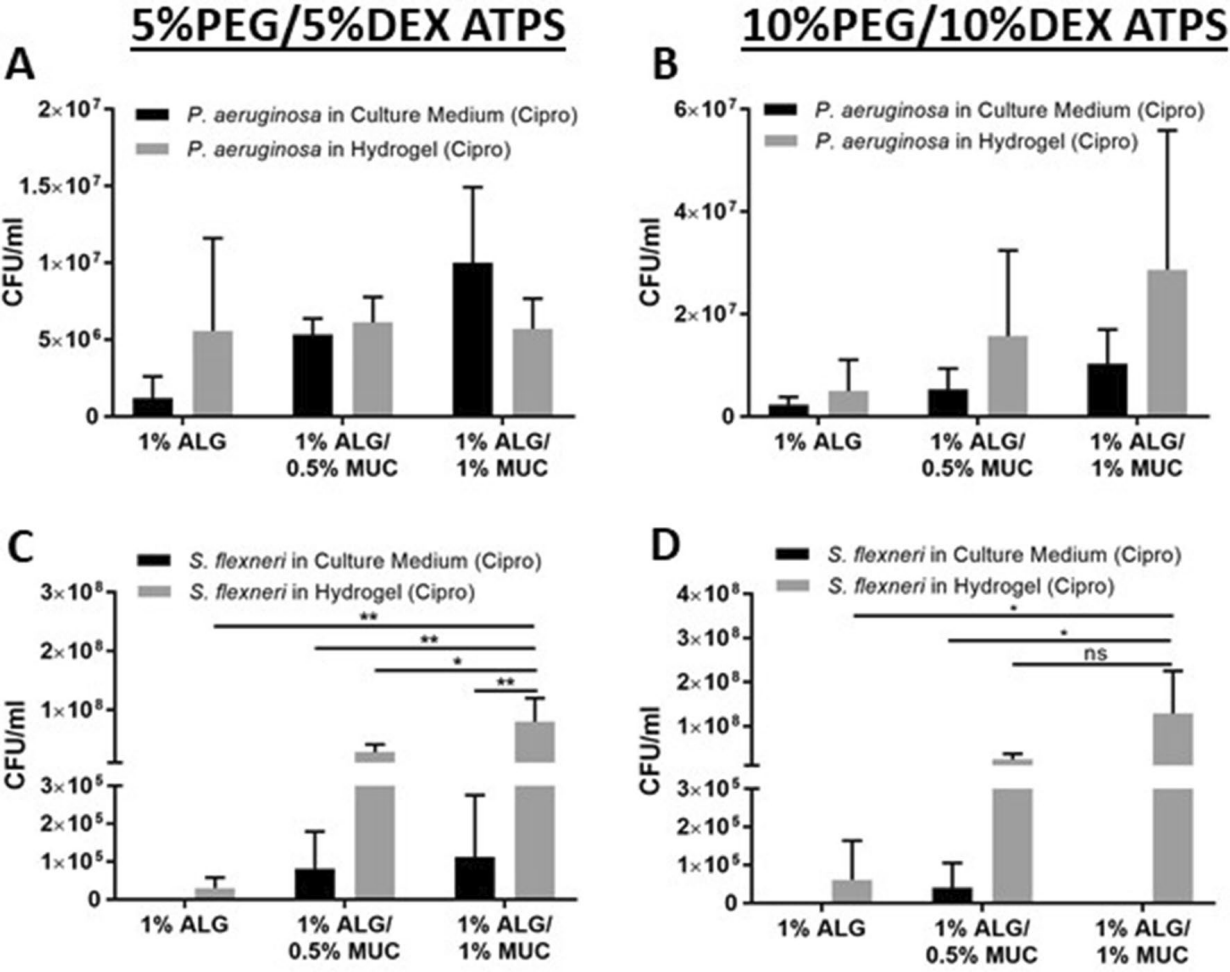
*P. aeruginosa* and *S. flexneri* abundance (CFU/ml) within the culture medium or hydrogel layer after 12 h of co-culture with 16-HBE and Caco-2, respectively, where PEG-phase media was supplemented with ciprofloxacin (0.125 μg/ml). CFU/ml values were calculated from *P. aeruginosa* grown in either a 5%PEG/5%DEX ATPS (**A**) or 10%PEG/10%DEX ATPS (**B**), and *S. flexneri* in a 5%PEG/5%DEX ATPS (**C**) or 10%PEG/10%DEX ATPS (**D**), where *indicates *p* < 0.05, **indicates *p* < 0.01, and ns = non-significant (n = 3).

After exposure to the antibiotic for 12 h, there were no significant differences found in *P. aeruginosa* abundance between the different hydrogels used or between the hydrogel layer and the culture medium (Fig. 2A,B). However, the presence of the antibiotic was able to reduce the overall abundance of *P. aeruginosa* in both the 5% PEG/5% DEX and 10% PEG/10% DEX ATPS co-cultures, such that the abundance was found to be higher in the hydrogel layer of the 1% ALG hydrogel compared to the culture medium of the 5% PEG/5% DEX ATPS (Fig. 2A,B). In the 10% PEG/10% DEX ATPS, on the other hand, there was an increase in bacterial abundance within the hydrogel layer with increasing mucin content (Fig. 2B).

The antibiotic supplementation was found to be most effective against *S. flexneri*, in both 5% PEG/5% DEX and 10% PEG10% DEX ATPS co-cultures. With the addition of a PEG-phase supplemented with ciprofloxacin, *S. flexneri* was fully eradicated in the culture medium of the co-cultures containing a 1% ALG hydrogel (Fig. 2C,D). Bacteria within the culture medium of co-cultures containing ALG-MUC hydrogels persisted but were reduced in comparison to no antibiotic treatment. The *S. flexneri* that persisted within the hydrogel were found to be significantly higher in abundance within hydrogels of increasing mucin concentrations (Fig. 2C,D). Altogether, these findings suggest that the presence of mucin within the bacterial microenvironment aids in both proliferation and antibiotic resistance of pathogenic bacterial species.

### Regional cell viability of mammalian cells when co-cultured with *P. aeruginosa *and *S. flexneri*

**Figure 3:**
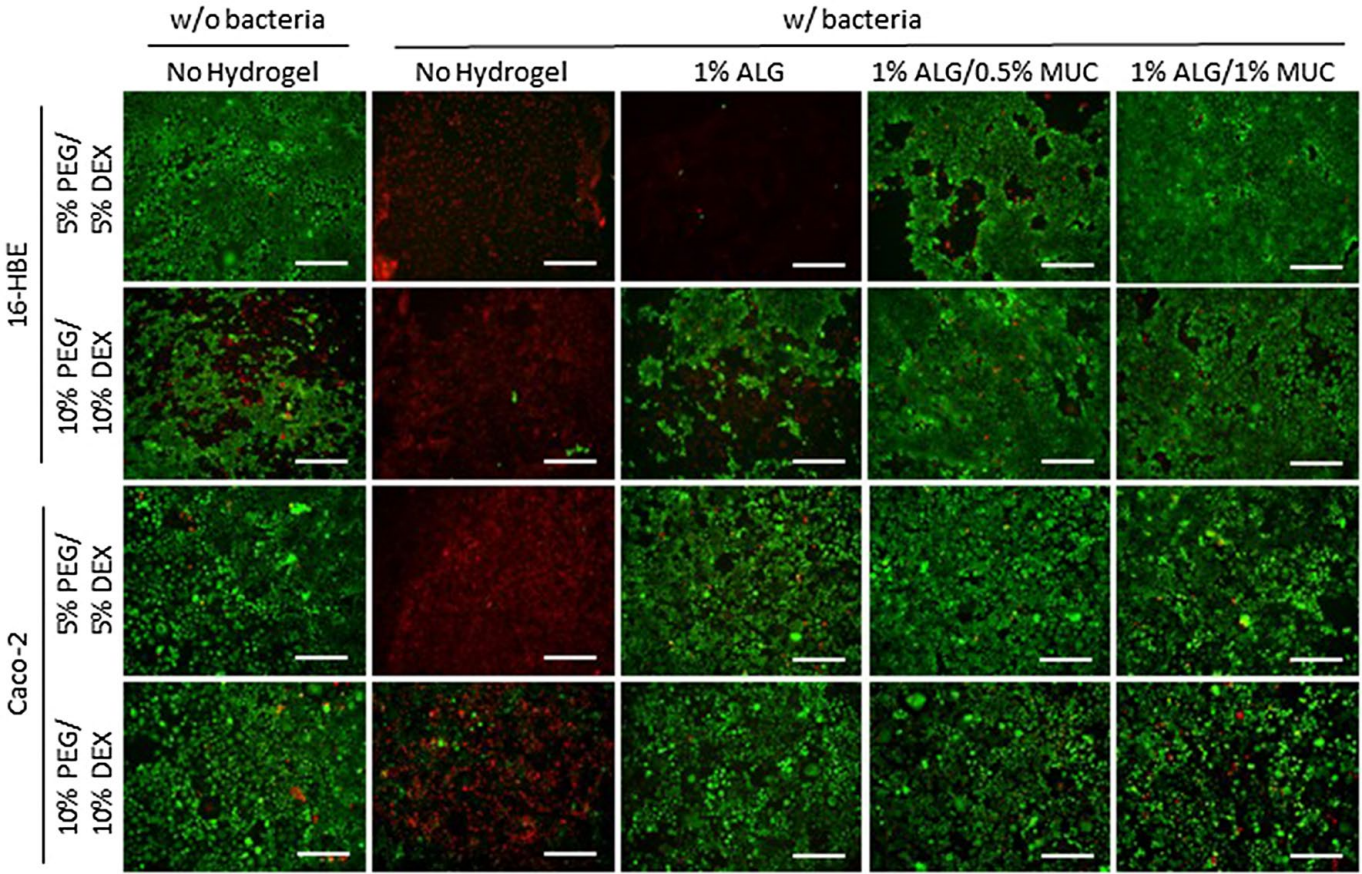
Live/Dead staining images of 16-HBE and Caco-2 monolayers after a 12-h co-culture with *P. aeruginosa* and *S. flexneri*, respectively, deposited with a PEG-DEX ATPS either with or without alginate-based hydrogels between mammalian cells and ATPS. Bacteria were deposited using a 5% PEG/5% DEX ATPS or 10% PEG/10% DEX ATPS over mammalian cells with an alginate-based hydrogel (1% ALG, 1% ALG/0.5% MUC, and 1% ALG/1% MUC) overlay, where a DEX droplet with or without bacteria was deposited directly onto mammalian cells as controls (scale bar = 500 μm).

After the 12-h incubation of each mammalian–microbial co-culture, the underlying mammalian cells were assessed by performing a Live/Dead assay, where all images were taken directly underneath the area of the DEX droplet (the area of highest bacterial density). For both cell lines (16-HBE and Caco-2), two control wells were used where an ATPS was deposited either with or without bacteria suspended in the DEX droplet directly on top of the mammalian cells. In co-cultures without a hydrogel layer between the mammalian and bacterial cells, both cell lines displayed the highest level of cell death within the area of the DEX droplet. When 16-HBE cells were co-cultured with *P. aeruginosa*, the level of cell death was found to be negatively correlated with increasing mucin concentrations within the alginate-based hydrogels, for both the 5% and 10% PEG-DEX ATPS (Fig. 3). Caco-2 cells, on the other hand, showed higher proportions of live cells when *S. flexneri* were deposited on top of all hydrogel formulations (Fig. 3).

**Figure 4:**
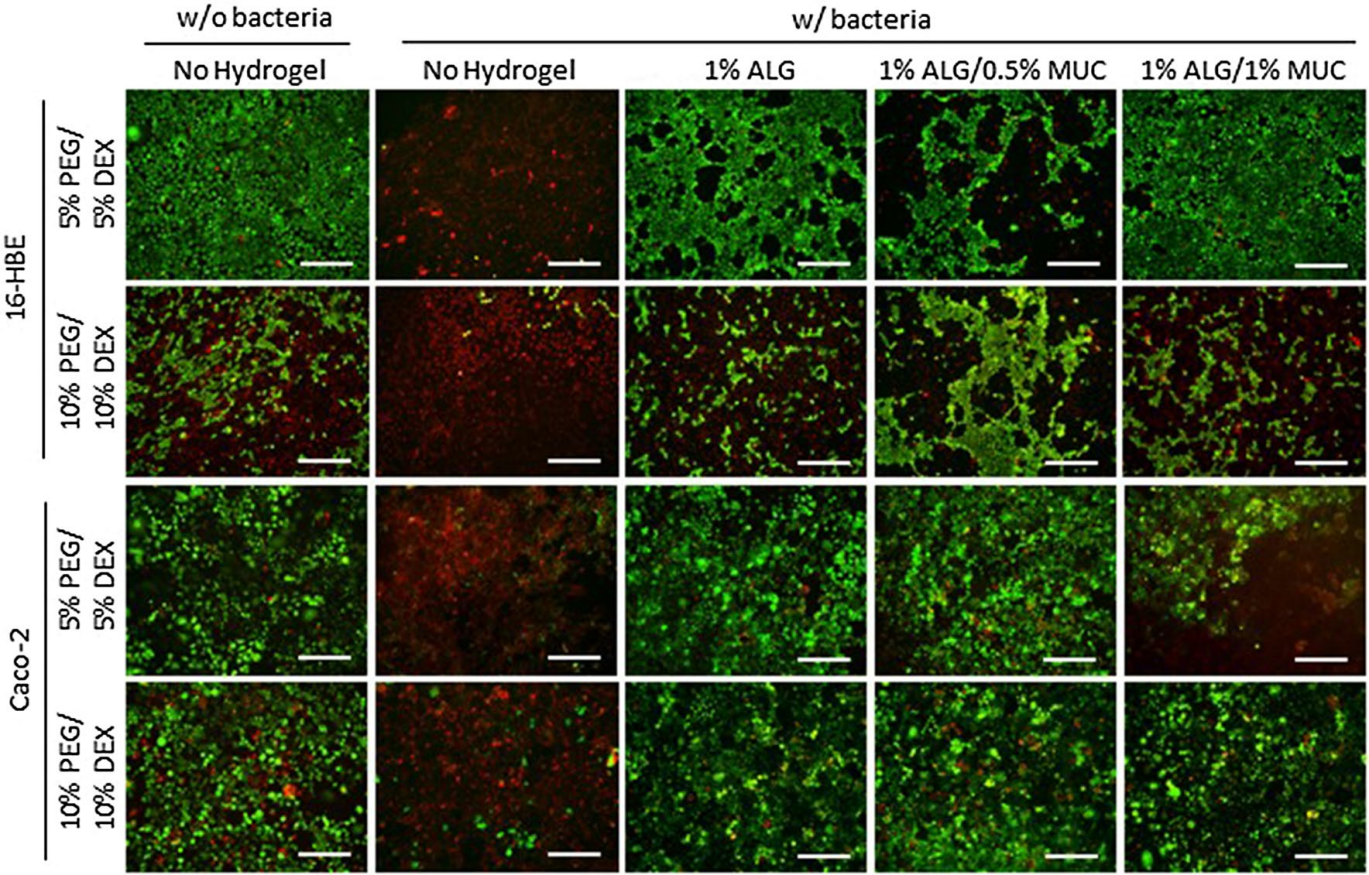
Live/Dead staining images of 16-HBE and Caco-2 cells after a 19-h (with ciprofloxacin supplemented PEG-phase added after 7 h) co-culture with *P. aeruginosa* and *S. flexneri*, respectively, deposited with a PEG-DEX ATPS either with or without an alginate-based hydrogel between mammalian cells and ATPS. Bacteria were deposited using a 5% PEG/5% DEX ATPS or 10% PEG/10% DEX ATPS over mammalian cells with an alginate-based hydrogel (1% ALG, 1% ALG/0.5% MUC, and 1% ALG/1% MUC) overlay, where a DEX droplet with or without bacteria was deposited directly onto mammalian cells as controls (scale bar = 500 μm).

With the introduction of the ciprofloxacin supplemented PEG-phase, the survival of 16-HBE cells seemed to be improved in co-cultures containing a 1% ALG hydrogel overlay (5% and 10% PEG-DEX ATPS). However, 16-HBE cell viability seemed to be less than what was observed when no antibiotic was supplemented in co-cultures containing ALG-MUC hydrogel (1% ALG/0.5% MUC and 1% ALG/1% MUC) overlays (Fig. 4). Similarly, this was observed for Caco-2 cells grown in a 5% PEG/5% DEX ATPS but not the 10% PEG/10% DEX ATPS, where Caco-2 cells showed similarly high levels of live mammalian cells cultured under all hydrogel formulations (Fig. 4).

**Figure 5:**
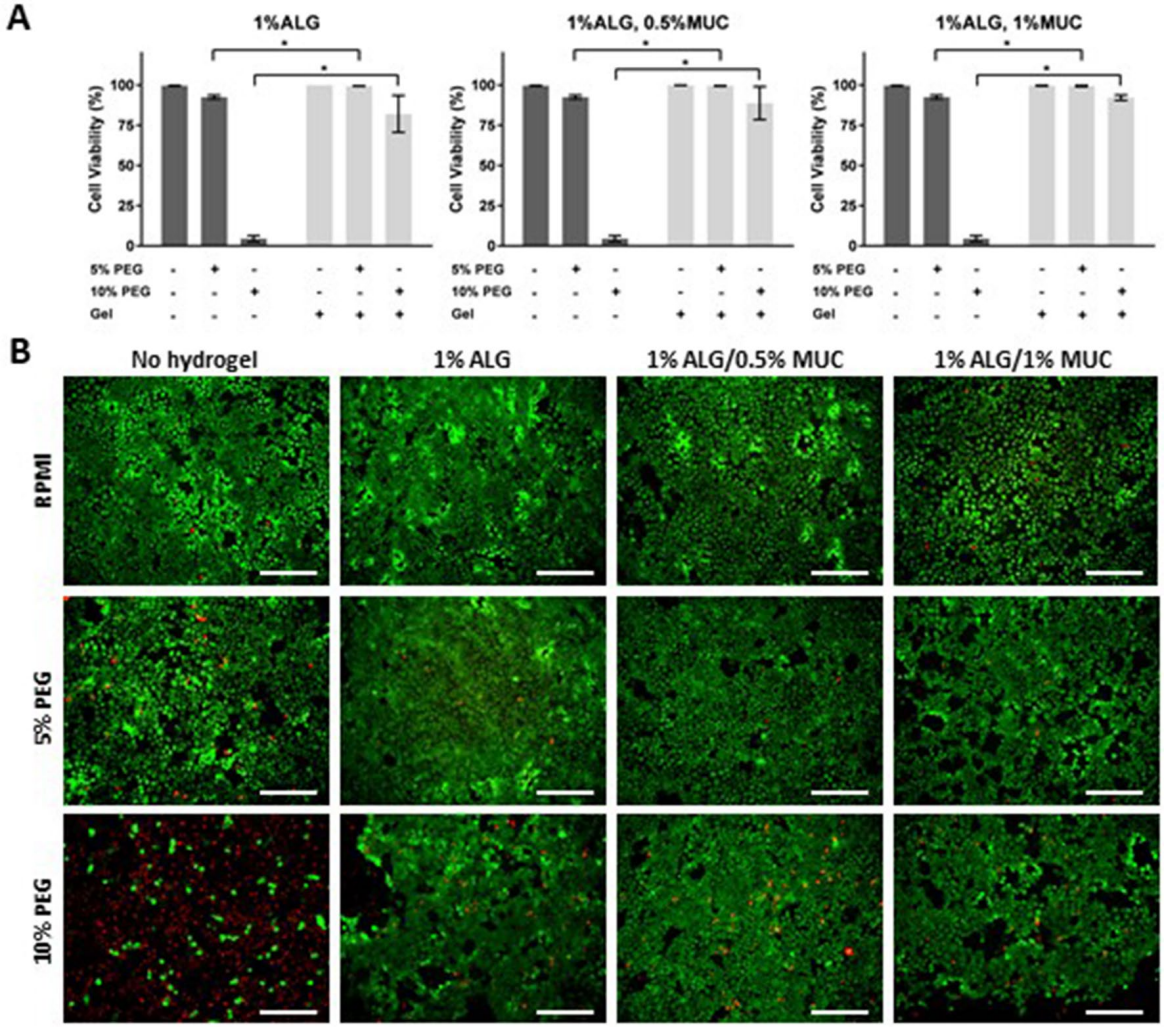
Mitigation of PEG-mediated cytotoxicity in 16-HBE cells through the addition of a 1% ALG and mixed ALG-MUC hydrogel layers. (**A**) Cell viability of 16-HBE cells with and without alginate-based hydrogel (1% ALG, 1% ALG/0.5% MUC, and 1% ALG/1% MUC) layers between mammalian cells and PEG-phase solution, where *indicates *p* < 0.05 (n = 3). (**B**) Fluorescent images of Live/Dead stained 16-HBE cells exposed to 5% and 10% PEG-phase solution with or without an alginate-based hydrogel (1% ALG, 1% ALG/0.5% MUC, and 1% ALG/1% MUC) overlay (Scale bar = 500 μm).

Polyethylene glycol has previously been shown to have cytotoxic effects towards mammalian cells in a molecular weight dependent manner^18^. Therefore, to demonstrate that the observed cell death was not due to the PEG or the alginate-based (1% ALG, 1% ALG/0.5% MUC, and 1% ALG/1% MUC) hydrogels, 16-HBE cells were incubated with a hydrogel overlay in either normal culture media (RPMI) or a PEG-rich phase (5% and 10%) (Fig. 5A,B). The resulting cell viability, after 48 h of incubation, demonstrated that all hydrogel formulations were able to significantly reduce cell death, under 5% and 10% PEG-phase conditions compared to when no hydrogel was present (Fig. 5A).

### ATPS stability is improved on higher mucin content hydrogels

**Figure 6:**
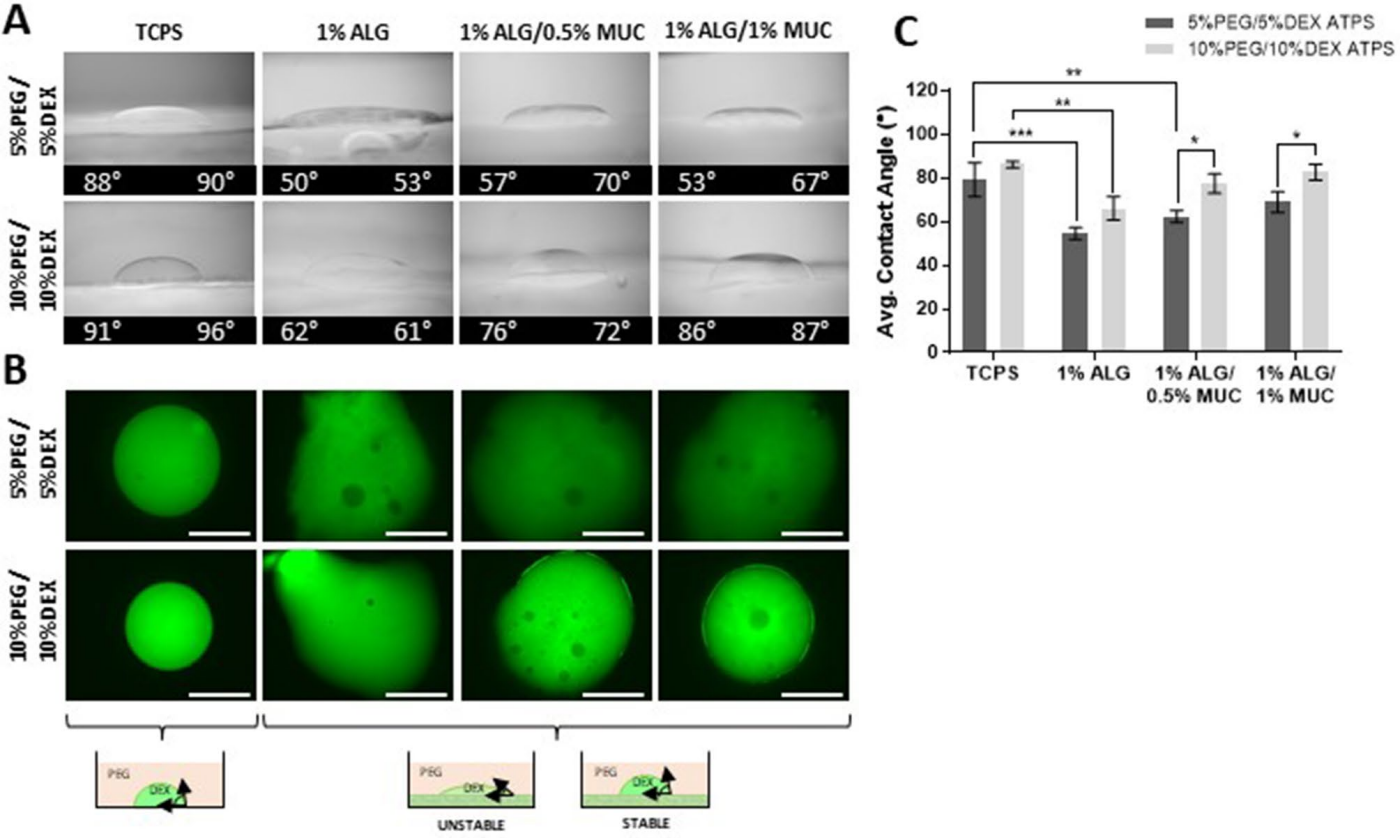
PEG-DEX ATPS formation on top of alginate-based hydrogel surfaces. (**A**) Side view images of DEX droplets formed on tissue culture polystyrene (TCPS), or alginate-based hydrogels surfaces (1% ALG, 1% ALG/0.5% MUC, 1% ALG/1% MUC) with left and right contact angle measurements. (**B**) Fluorescent images of FITC-DEX droplets formed within a PEG-DEX ATPS on top of TCPS, or alginate-based hydrogel surfaces (Scale bar = 1 mm). (**C**) Average contact angles of DEX droplets formed within a PEG-DEX ATPS on top of the different surfaces, where *indicates *p* < 0.05, **indicates *p* < 0.01, and ***indicates *p* < 0.001 (n = 3).

The DEX droplet contact angle is an important measure, as it is an indicator of the interfacial tension between the two liquid phases (DEX and PEG), where higher contact angles suggest higher interfacial tension and more stable droplets^22^. Therefore, it was hypothesized that the higher abundance of bacteria within the culture medium of co-cultures containing 0.5% and 1% mucin hydrogel was due to poor bacterial containment as a result of lowered interfacial tension. However, DEX droplet contact angles were found to increase with increasing mucin concentrations within the hydrogel (Fig. 6A,C). We further illustrated this by imaging a FITC-DEX droplet on top of the different alginate-based hydrogel surfaces, where a tissue culture-treated polystyrene (TCPS) surface was used as a control. We found that DEX droplets were dispersed and less circular on 1% ALG and 1% ALG/0.5% MUC hydrogels suggesting increased contact angle and interfacial tension when a PEG-DEX ATPS is formed on higher mucin concentration hydrogels (Fig. 6B).

### Increased mucin reduces elastic characteristic while increasing viscous characteristic and does not affect molecular diffusion of alginate-based hydrogels

**Figure 7:**
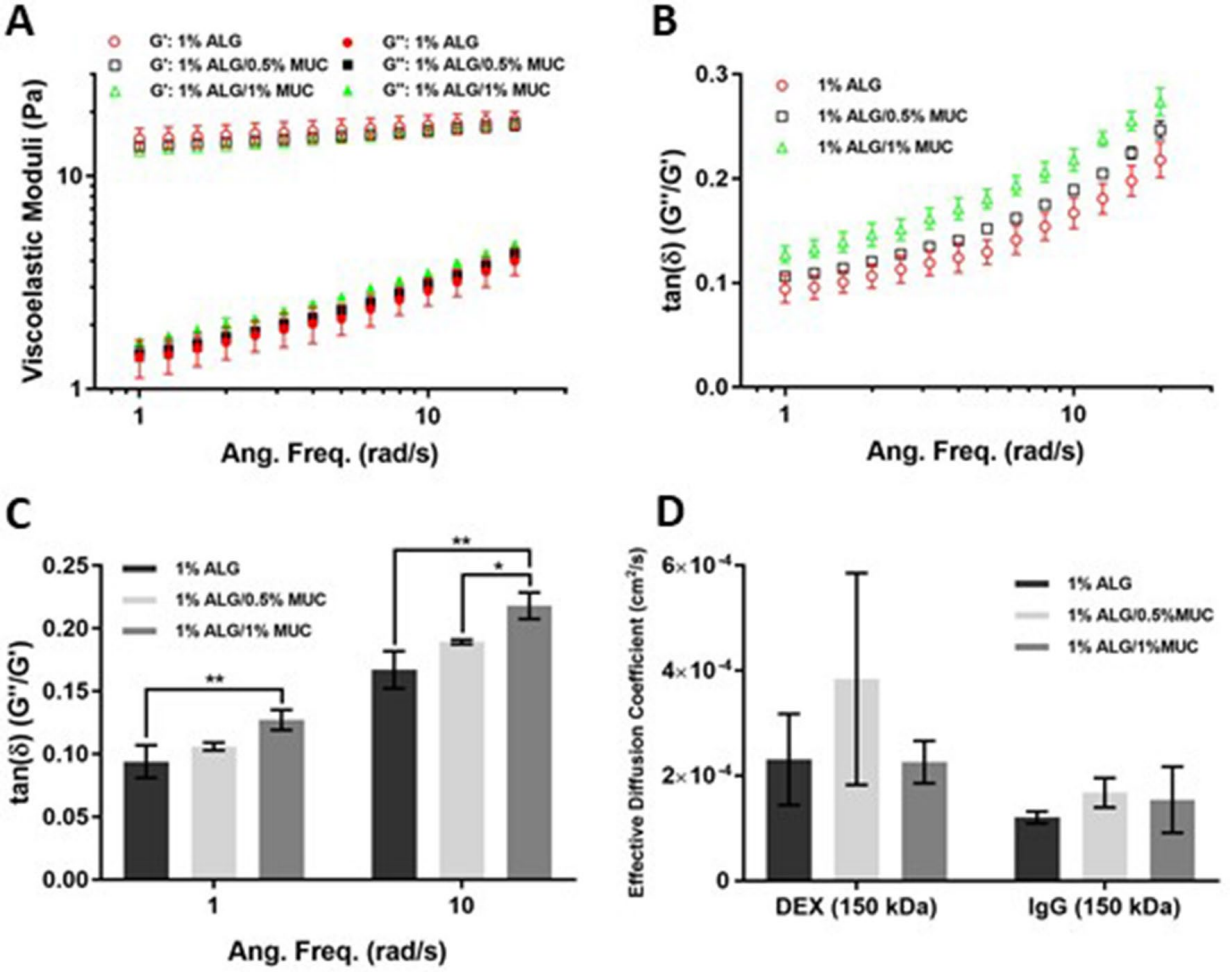
Rheological measurements and effective diffusivity of alginate-based hydrogels (1% ALG, 1% ALG/0.5% MUC, and 1% ALG/1% MUC). (**A**) Viscoelastic moduli of alginate-based hydrogels measured at angular frequencies from 1 to 19.95 rad/s, where G′ is the storage (elastic) modulus and G″ is the loss (viscous) modulus. Loss tangent of alginate-based hydrogels at a range of angular frequencies (1–19.95 rad/s) (**B**) and a comparison of tan(δ) at low (1 rad/s) and high (10 rad/s) angular frequencies, where *indicates *p* < 0.05 and **indicates *p* < 0.01 (n = 3). (**D**) Effective diffusion coefficients (D_eff_) of dextran (DEX) and immunoglobulin G (IgG) within alginate-based hydrogels (n = 3).

The viscoelastic properties of the mucus layer can affect both molecular diffusion and microbial mobility within the mucus-epithelium niche^23^. Therefore, we sought to measure the rheological properties of the alginate-based hydrogels using a plate rheometer. Both the storage (elastic, G′) modulus and the loss (viscous, G″) modulus were measured, where we found the elastic characteristic (G′) was dominant over the viscous characteristic (G″) for all hydrogel formulations (1% ALG, 1% ALG/0.5% MUC, and 1% ALG/1% MUC) (Fig. 7A). To better understand the relationship between G′ and G″, ratios of G″:G′ (tan(δ), loss tangent) were calculated at each angular frequency (1–19.95 rad/s). The tan(δ) was found to be consistently higher in the 1% ALG hydrogels compared to the mixed ALG-MUC hydrogels (1% ALG/0.5% MUC and 1% ALG/1% MUC), indicating a reduction in elastic characteristic and an increase in viscous characteristic as mucin concentrations are increased (Fig. 7B).

At high angular frequency (10 rad/s), tan(δ) of the 1% ALG/1% MUC hydrogel was significantly higher than both the 1% ALG and 1% ALG/0.5% MUC hydrogels. Whereas, at a low angular frequency (1 rad/s), tan(δ) of the 1% ALG/1% MUC was only found to be significantly higher than the 1% ALG hydrogel (Fig. 7C).

Interestingly, diffusivity seemed to be unaffected by the differences in viscoelasticity. The effective diffusion coefficients (D_eff_) of DEX and IgG did not show any significant differences between the three alginate-based hydrogels (1% ALG, 1% ALG/0.5% MUC, and 1% ALG/1% MUC) (Fig. 7D). Although non-significant, the D_eff_ of DEX in a 1% ALG/0.5% MUC hydrogel was found to be greater compared to the D_eff_ in a 1% ALG hydrogel and a 1% ALG/1% MUC hydrogel. IgG, on the other hand, showed greater D_eff_ values in the mucin containing hydrogels (1% ALG/0.5% MUC and 1% ALG/1% MUC) compared to the 1% ALG hydrogel (Fig. 7D).

## Discussion

The human mucus layer acts as the first line of defense against any pathogens or foreign particles that may enter the body^1^. However, some bacterial species have adapted to bypass the mucus layer, while other opportunistic bacteria will cause infection and inflammation when the mucus layer is compromised due to dysregulation^24,25^. While animal models remain to be the standard for studying host–pathogen interaction^26^, in vitro disease modeling may serve as a valuable tool to validate findings in animal studies, especially considering that findings from animal models do not always align with clinical observations^27,28^. To model host–pathogen interactions in vitro, it is crucial to have controlled bacterial growth and to provide the proper microenvironment suitable for both mammalian and bacterial proliferation. Many of the current mammalian–microbial co-culture platforms have demonstrated stable growth of up to 7 days, under flow conditions^14,29^, but are complex and difficult for the average life science laboratory to implement. Other techniques lack a sufficient mucus microenvironment for these interactions to take place in a physiologically relevant manner^11,13,30^. Here, we demonstrate the use of a simple and robust mammalian–microbial co-culture method using a PEG-DEX ATPS with the incorporation of a mixed ALG-MUC hydrogel to recapitulate the 3-dimensional mucus microenvironment.

Although PEG has been known to be cytotoxic towards mammalian cells, the use of a PEG-DEX ATPS for mammalian–microbial co-culture should not be disregarded considering its superior phase separation properties, which allow for bacterial confinement and controlled biofilm formation^13,31,32^. The mechanism of PEG-mediated cell death is primarily due to its ability to interrupt cellular membranes and cause membrane fusion between cells and generation of reactive oxygen species (ROS)^18,33^. However, with the incorporation of a mixed ALG-MUC hydrogel, we demonstrated its compatibility with a PEG-DEX ATPS by mitigating the PEG-mediated cytotoxic effects. PEG-mediated cell death was significantly reduced while allowing for sufficient nutrient and oxygen diffusion to maintain cell viability by overlaying the mammalian cells with both the 1% ALG and mixed ALG-MUC (1% ALG/0.5% MUC and 1% ALG/1% MUC) hydrogels. This enables the use of the ATPS bacterial deposition technique on top of the engineered mucus-like hydrogel layer, while ensuring the survival of the underlying mammalian cells.

Another important factor to consider when using a PEG-DEX ATPS for bacterial deposition and co-culturing techniques is the ability to confine the bacterial colony within the DEX droplet. The contact angle at the PEG-DEX interface with the surface at which it is deposited is a function of the interfacial tension between the two phases and is an important determinant of the containment function of an ATPS^34^. The DEX droplet contact angles measured on the ALG-MUC hydrogel surfaces were found to be similar to those of DEX droplets measured when deposited onto a tissue culture polystyrene surface, where only the 1% ALG hydrogel surface was found to produce a significantly lower contact angle. This suggested that with the addition of the mucin to the alginate hydrogels, larger contact angles were produced and thus could allow for a stronger barrier function of the DEX in containing the bacteria to the given region of the droplet. This phenomenon can be explained by the presence of lipids within the crude mucin mixture creating a more hydrophobic surface for ATPS formation leading to greater DEX droplet contact angles^35,36^. Although this finding was unexpected, it was found to be advantageous for the PEG-DEX ATPS co-culture platform.

Changes in the ALG-MUC hydrogel formulations can also affect the physical properties, such as viscoelasticity and diffusivity. As previously described by Mezger (2020), the viscoelastic moduli of a gel-like material, G″ and G′, represent the solution (sol) and gel components, respectively^37^. Natural mucus is known to display non-Newtonian viscoelastic properties and shear thinning, such that under low shear rates mucus will behave more gel-like and under high shear rates mucus will show more sol-like characteristics^38,39^. Our findings showed that mixed ALG-MUC hydrogels had higher values of tan(δ) (G″/G′), indicating that higher mucin concentrations within the alginate hydrogels led to a higher G″. Considering that G′ was relatively similar between hydrogel formulations and consistent within the range of angular frequencies (1–19.95 rad/s), this suggests that the stored energy within the hydrogel network remained the same while loss energy was increased when mucin concentrations were increased. It is possible that there is a proportion of the incorporated mucins that freely move within the hydrogel network without interaction, causing an increase in G″. Alternatively, mucins are known to contain negatively charged carbohydrate sidechains that may sequester the calcium cations and lead to other potential crosslinking interactions such as ALG-MUC or MUC-MUC interaction^3,40^, thus reducing the level of crosslinking and increasing G″. We also acknowledge that calcium concentrations remained constant as mucin concentrations were increased which may have also contributed to the increase in G″.

We were also interested in measuring the diffusivity of the alginate-based hydrogels to determine whether they were affected by the differences in viscoelasticity. Although non-significant, D_eff_ of IgG was found to be higher in the mixed ALG-MUC hydrogels compared to the 1% ALG formulation. This finding is consistent with the higher sol-like characteristic of mixed ALG-MUC hydrogels potentially resulting in greater molecular mobility^37^. However, this is inconclusive considering the lack of significance in the diffusivity data.

Following the fabrication and physical characterization of mixed ALG-MUC hydrogels, we next aimed to establish a mammalian–microbial co-culture system using a PEG-DEX ATPS with the engineered mucus overlay. Here, we matched 16-HBE cells with a common airway pathogen, *P. aeruginosa*, and Caco-2 cells with *S. flexneri*, a pathogen known to infect the colonic region of the GI tract. For *P. aeruginosa*, our findings demonstrate that the bacteria preferentially grow within the hydrogel layer when using 1% ALG hydrogels as opposed to the mixed ALG-MUC hydrogels (1% ALG/0.5% MUC and 1% ALG/1% MUC), where bacterial abundance was found to be higher in the culture medium compared to the hydrogel layer. This preferential growth between hydrogels with and without mucin may have contributed to the higher levels of mammalian cell death in 16-HBE cells overlaid with a 1% ALG hydrogel, simply by being in closer proximity to the cells. *Pseudomonas* *aeruginosa* are known to secrete toxins into the extracellular environment^41^, thus concentrating the harmful secretions in the local region. We must also consider the differences in solid content between hydrogels with and without mucin, where these secreted toxins needed to diffuse through denser polymer networks in mixed ALG-MUC hydrogels as opposed to the 1% ALG hydrogels. *S. flexneri*, on the other hand, were found to consistently favor the hydrogel layer. Since *S. flexneri* are non-flagellated, they would be less likely to appear in the culture medium after integrating into the hydrogel layer. Additionally, *Shigella* *flexneri* have been previously found to bind to mucin in which mucin carbohydrate side chains can be used by the bacteria as a nutrient source to benefit colony expansion within the mucus layer^42^.

With the introduction of ciprofloxacin at minimum inhibitory concentrations, the bacteria escaping the DEX droplet into the PEG-phase was reduced for *P. aeruginosa* and nearly eradicated for *S. flexneri*. However, bacteria that were able to migrate into the hydrogel layer seemed to be protected from antibiotic. Some potential mechanisms of antibiotic resistance that have been previously proposed are (1) antibiotic binding to the mucin thus reducing the availability within the microenvironment, or (2) alterations in bacterial physiology to allow for increased antibiotic resistance^21,43^. Moreover, *P. aeruginosa* are notorious for their infectivity in CF patients and resistance to antibiotics through dense biofilm formation^10,44^. This infectivity and persistence has been known to come from the ability to undergo changes in gene expression to shift into a mucoid state where flagella protein expression is reduced and alginate production is increased to form dense biofilms, which can limit the diffusion of antibiotics through the extracellular polymeric substances of the biofilm^45,46^. In diseases such as CF, persistent bacterial infection remains to be a life-threatening issue for patients and continues to be investigates with both in vivo and in vitro methods^47,48^.

Although the hydrogels used in this study were simplistic, it allows for ease of use and a robust mucus-like hydrogel to mimic the biochemical properties of natural mucus. In nature, prior to mucin secretion, the mucin is tightly packaged into secretion granules within epithelial and goblet cells and once released into the extracellular environment, the mucin electrolyte content becomes diluted leading to swelling and the formation of a mucus hydrogel^49^. In contrast, once commercially processed mucins become dehydrated during purification, where rehydration of the mucin does not result in the reformation of a viscoelastic hydrogel, as previous work has shown^50^. While purified and commercially available mucin has been used to generate mucus hydrogels previously using PEG-thiol crosslinkers, these techniques required long gelation times of up to 15 h^51^. The mixed ALG-MUC hydrogels used in this study can be formed directly on top of mammalian cells within an hour of gelation and provide the biochemical properties of mucin. We demonstrated that these hydrogels are compatible with a PEG-DEX ATPS co-culture in that bacteria can be deposited directly on top of the engineered mucus layer for localized growth within the co-culture wells, while providing the underlying mammalian cells protection against PEG-mediated cytotoxicity. Another potential use of these ALG-MUC hydrogels is in testing drug diffusion as well as antibiotic treatments of opportunistic pathogens commonly found within the mucus microenvironment, in which conventional drug diffusion testing tends to neglect the steric and chemical interaction of drugs with the mucus barrier^52^. Nonetheless, ALG-MUC hydrogels containing varying mucin and crosslinking concentrations should be characterized to explore the tunability of the engineered mucus layer to further improve upon the mammalian–microbial co-culture system proposed in this study. Moreover, the bacterial biofilms within the hydrogel layer should also be characterized in terms of distribution and gene expression to understand the physiological state of the bacteria cells.

## Conclusions

The ALG-MUC hydrogels presented in this study were able to maintain cell viability and were compatible with a PEG-DEX ATPS mammalian–microbial co-culture. We demonstrate that an overlay of alginate-based hydrogel on top of a mammalian monolayer can mitigate the cytotoxic effects of PEG thus allowing for the use of PEG-DEX ATPS formulations. Moreover, the incorporation of mucin into the alginate-based hydrogels allows for improved ATPS stability, when deposited on the hydrogel surface. We also demonstrated that increasing mucin concentrations within the alginate-based hydrogels led to more sol-like properties while elastic characteristic remained relatively consistent. Additionally the viscoelastic moduli were found to fall within a similar viscoelastic range as natural mucus samples^39,53^. The diffusivity, on the other hand, was unaffected by the incorporation of mucin. When the hydrogels were incorporated into the PEG-DEX ATPS mammalian–microbial co-cultures, we demonstrate the important role of mucin such that the presence of mucin within the alginate-based hydrogels provide improved protection from pathogenic bacteria as well as a microenvironment for pathogenic bacteria to persist in the presence of antibiotics. Overall, the findings in this study demonstrates the use of a simple and robust mammalian–microbial co-culture system, which can be used to better understand host–pathogen interaction by providing a realistic mucus microenvironment.

## Materials and methods

### Mammalian cell culture

Cell lines used in this study include a human bronchial epithelial cell line, 16-HBE (kindly provided by Dr. Elizabeth Cowley, Dalhousie University), and a colorectal adenocarcinoma cell line, Caco-2 (kindly provided by Dr. Andrew Stadnyk, Dalhousie University). 16-HBE cells were grown and maintained in DMEM/F-12 media, a 1:1 mixture of Dulbecco’s Modified Eagle Medium (DMEM, Gibco) and Ham’s F-12 Nutrient Mixture (Gibco), supplemented with 10% (v/v) fetal bovine serum (FBS, Gibco) and 1% (v/v) antibiotic–antimycotic (anti-anti, 100x, Gibco) at 37 °C, 5% CO_2_. Caco-2 cells were grown and maintained in DMEM (10% FBS, 1% anti-anti) at 37 °C, 5% CO_2_.

### Bacteria cell culture conditions

Bacterial strains used include *Pseudomonas aeruginosa* PA14 and *Shigella flexneri* M90T (gifted by Dr. Zhenyu Cheng, Dalhousie University). *P. aeruginosa* cultures were grown in LB broth (Sigma-Aldrich) supplemented with 250 μg/ml carbenicillin disodium salt (Sigma-Aldrich) and incubated at 37 °C with shaking at 200 rpm. All cultures were grown from single colonies after streaking LB agar (1.5%, w/v, Sigma-Aldrich) plates (250 μg/ml carbenicillin) from frozen stocks.

*Shigella* *flexneri* cultures were grown in tryptic soy broth (TSB, Sigma-Aldrich) supplemented with 100 μg/ml ampicillin (Sigma Aldrich) and incubated at 37 °C with shaking at 200 rpm. All cultures were grown from single colonies after streaking TSB Congo red (200 μg/ml, Sigma-Aldrich) agar (1.5%, w/v) plates (100 μg/ml ampicillin) from frozen stocks.

### Preparation of aqueous two-phase systems

Aqueous two-phase system (ATPS) formulations were prepared at 5% and 10% (w/v) polyethylene glycol (PEG, 35 kDa, Sigma-Aldrich) and dextran (DEX, 500 kDa, Pharmacosmos) in either RPMI medium or DMEM containing 1% (v/v) FBS (Thermo Fisher Scientific) and 1% (v/v) anti-anti (F1 and F2). Anti-anti was only used for PEG-cytotoxicity assays when bacteria was not being introduced into the cell culture, where ATPS formulations used for co-cultures did not contain anti-anti (F3 and F4). To make the ATPS, PEG and DEX were dissolved in cell culture medium (RPMI or DMEM) by mixing overnight in a 50 ml centrifuge tube on a rocking platform shaker (VWR) followed by addition of 1% (v/v) FBS and 1% (v/v) anti-anti, then sterilized by suction filtration (0.22 μm). Filter sterilized ATPS were centrifuged for 90 min at 3000×*g* to obtain phase separation of the PEG-phase (upper) and DEX-phase (lower). The two phases were then aliquoted into separate centrifuge tubes and stored at 4 °C.

### Alginate-mucin hydrogel fabrication

Hydrogels used in this study included three formulations consisting of alginate (Low Viscosity Alginic Acid Sodium Salt, MP Biomedical) and mucin (Mucin Type II, Sigma Aldrich): 1% alginate (1% ALG), 1% alginate with 0.5% mucin (1% ALG/0.5% MUC), or 1% alginate with 1% mucin (1% ALG/1% MUC), crosslinked using calcium chloride (CaCl_2_, Sigma Aldrich). Alginate was dissolved in distilled water (2% w/v) by mixing overnight using a magnet stir rod, filter sterilized (0.45 μm pore size), and then frozen at − 20 °C and lyophilized (FreezeZone 2.5 Liter Benchtop Freeze Dryer, Labconco). Lyophilized alginate was then exposed to UV radiation (250 nm wavelength) for one hour and reconstituted in PBS for a 3% (w/v) solution. Mucin was sterilized by spreading dry mucin on a petri dish and covering it with 95% ethanol, then incubating at 70 °C for 24 h. A 10% (w/v) mucin stock solution was then prepared by mixing in PBS. Hydrogels were fabricated by mixing the 3% alginate solution with the 10% mucin stock solution at calculated volumes to generate 1% (w/v) alginate and varying mucin concentrations (0%, 0.5%, or 1%; w/v) with added PBS to make up half of the total volume. For the remaining half of the total hydrogel mixture, an 11 mM CaCl_2_ (in DMEM) solution was added and carefully mixed to prevent air bubble formation a final CaCl_2_ concentration of 5.5 mM (See Table 1 for example volumes). Hydrogels were then incubated at 37 °C for 60 min to allow for gelation.

**Table 1:** Alginate and mixed alginate-mucin hydrogel volumes (μl) for 900 μl of hydrogel.

	Hydrogel formulation		
	1% ALG/0% MUC	1% ALG/0.5% MUC	1% ALG/1% MUC
3% alginate sol	300	300	300
PBS	150	105	60
10% mucin sol	0	45	90
CaCl_2_ (in DMEM)	450	450	450
Total vol	900	900	900

### Preparation of an ATPS mammalian–microbial co-culture containing alginate-mucin hydrogels

Two sets of mammalian–microbial co-cultures were carried out: 16-HBE cells with *P. aeruginosa* and Caco-2 cells with *S. flexneri*. First, each cell line was seeded into a 24-well plate at a cell density of ~ 4000 cells/well (for Caco-2) and ~ 8000 cells/well (for 16-HBE) then grown for 24 h followed by a media change to low FBS containing RPMI (Gibco) (1% FBS, 1% anti-anti) or DMEM (1% FBS, 1% anti-anti) for 16-HBE and Caco-2, respectively. Alginate and alginate-mucin hydrogels (300 μl) were deposited on top of the monolayers and incubated at 37 °C for 1 h to allow for hydrogel gelation. As hydrogels were incubating, *P. aeruginosa* and *S. flexneri* overnight cultures were resuspended in DEX-phase (F3 & F4) at a bacterial density of ~ 3 × 10^8^ CFU/ml. PEG-phase (200 μl, F3 & F4) was then carefully pipetted into each well containing hydrogels followed by deposition of 0.5 μl of the bacteria-rich DEX-phase, with the respective mammalian cells, using a Biomek 4000 (Beckman Coulter) liquid handling robot. The 24-well plate was then imaged for 12 h using an automated microscope (Evos FL Auto 2, Invitrogen) with an onstage incubator at 37 °C, 5% CO_2_ with 80% humidity.

Co-culture experiments involving the use of antibiotic were initially set up as mentioned above but incubated for 7 h to establish bacterial communities within the DEX droplet. After the 7-h incubation, the PEG-phase was carefully removed, using a pipette, and 200 μl of PEG-phase supplemented with 0.125 μg/ml ciprofloxacin (Sigma-Aldrich) was added slowly to avoid disrupting the bacterial communities formed on top of the hydrogels. The antibiotic treated co-cultures were then incubated for another 12 h under the same conditions. Live/Dead and Hoechst staining was carried out after both antibiotic treatment and non-antibiotic treatment experiments.

### Determination of bacterial abundance within the liquid and hydrogel components of the ATPS co-culture through colony forming unit counting

Bacterial distribution within the co-culture system was determined by performing a colony forming unit (CFU) count by single plate-serial dilution spotting (SP-SDS) as described by Thomas et al. (2015). Briefly, the culture medium (PEG-phase and DEX-phase) was removed first by slowly aspirating using a pipette and transferred into a microcentrifuge tube. Followed by the removal of the hydrogel layer using wide bore pipette tips and transferred into a microcentrifuge tube. Samples were then diluted with 500 μl of PBS and vortexed for 30 s per tube. Tubes were then centrifuged at 16,000×*g* for 10 min to pellet bacteria and resuspended in 200 μl (ATPS sample) or 300 μl (hydrogel sample) of PBS. Resuspended samples were then serial diluted in a 96-well plate (10^−2^–10^−9^). Diluted samples were then spot plated (20 μl) accordingly in each section of the agar plates and airdried for 10 min to be incubate at 37 °C, 5% CO_2_ for 16 h, as illustrated in Fig. S1. Colonies were counted the next day (countable range = 6–60 colonies) and CFU/ml was calculated.

### PEG-DEX ATPS contact angle measurements on alginate-based hydrogel surfaces

To assess the stability of the DEX droplets on the different alginate-based hydrogel surfaces the contact angles of the DEX droplets were measured. To measure the contact angle of DEX droplets, custom well plates were fabricated by using a hand-held rotary tool (Dremel Micro) to carve out four wells from a 24-well plate. Wells were carved out to have a front and back opening where glass cover slips were epoxied for a front and rear window with a plastic ledge at the front window to prevent meniscus formation (Fig. S2). Alginate and alginate-mucin hydrogels were fabricated as mentioned above and deposited into the custom wells until the volume was level with the plastic ledge. After 1 h of incubation at room temperature (RT), an ATPS was formed on each hydrogel surface and tissue culture polystyrene (TCPS) with a 2 μl DEX droplet and imaged from a sideview using a Trinocular Stereo Zoom Microscope (3.5–180X, AmScope). Contact angles were then estimated using the angle tool on ImageJ, where two angles were measured per DEX droplet and averaged.

Additionally, the DEX droplet dispersion was assessed by imaging a fluorescent DEX droplet on the different alginate-based hydrogels surfaces. To generate the fluorescent DEX-phase, 15 μl of a 1% (w/v) FITC-DEX (150 kDa) was added to 300 μl of either a 5% or 10% DEX-phase. Alginate-based hydrogels (1% ALG, 1% ALG/0.5% MUC, and 1% ALG/1% MUC) were formed in 24-well plates using 300 μl of hydrogel and 200 μl of PEG-phase deposited on top of the hydrogel. The fluorescent DEX was deposited (2 μl) into the PEG-phase using a liquid handling robot (Biomeck 4000, Beckman Coulter) and allowed to settle on the hydrogel surface for 5 min.

### Cell viability with hydrogels and aqueous two-phase system components

To assess the ability of alginate-based hydrogels in mitigating PEG-mediated cytotoxicity, 16-HBE cells were seeded into a 24-well plates as mentioned previously. Media was then removed from each well and cells were washed once with PBS followed by the addition of 1% ALG or ALG-MUC (1% ALG/0.5% MUC or 1% ALG/1% MUC) hydrogels. PEG-phase (F1 or F2) or RPMI media (1% FBS and 1% anti-anti) was then added on top of the hydrogels and incubated at 37 °C, 5% CO_2_ for 48 h. After the incubation period, media and hydrogels were removed from the wells by suction and Live/Dead Assay (Thermo Fisher Scientific) and Hoechst (Thermo Fisher Scientific) stain were performed to assess cell viability. Calcein AM and ethidium homodimer-1 working concentrations were both 4 × 10^−4^ mM, while Hoechst was used at 5 μg/ml (8.1 × 10^–3^ mM), all diluted in PBS. Cell staining was carried out by removing growth media, where cells were washed with PBS, once. Live/Dead stain (0.2 ml, calcein AM and ethidium homodimer-1) was added to each well and incubated for 20 min at 37 °C, followed by a PBS wash. Hoechst stain (0.5 ml) was added to each well and incubated at RT for 10 min followed by 2 PBS wash steps and imaged using an epifluorescent microscope (EVOS FL Auto 2, Invitrogen).

After Live/Dead staining, fluorescent microscopy was performed where 5 images were taken per well using DAPI (nuclei), GFP (live cells), and RFP (dead cells) light filters. Each image was analyzed using Celleste Image Analysis Software to count the number of nuclei and dead cells. Cell viability was calculated by the following formula: Cell Viability (%) = (Tot. # cells—Dead cells/Tot. # of cells) × 100.

### Determination of alginate-based hydrogel viscoelastic parameters

Alginate-based hydrogels were formulated as described above, making a total volume of 1 ml in 1.5 ml centrifuge tubes. A controlled-stress/controlled-rate rheometer (AR2000, TA Instruments) was used to measure the viscosity, storage (elastic, G′) modulus, and loss (viscous, G″) modulus of the hydrogels. A 2°/40 mm cone-and-plate geometry was used, and the temperature was maintained at 23 °C by the Peltier plate. To reduce hydrogel evaporation, a solvent trap was also used.

For all tests, 590 µl of hydrogel was deposited directly onto the Peltier plate. Tests were initiated 15 min after deposition to allow the hydrogels to crosslink. Strain sweep measurements from 0.1 to 1000% strain were first performed at an angular frequency of 1 rad/s to determine the linear viscoelastic region of each hydrogel. To obtain the viscosity, storage modulus, and loss modulus of the hydrogels, frequency sweep measurements from angular frequencies of 0.1–100 rad/s were then performed at strain amplitudes found to be within the linear viscoelastic region (1–2% strain).

### Molecular diffusion through hydrogels by fluorescent imaging

Diffusion coefficients of fluorescein tagged DEX and IgG were assessed by fluorescent imaging over time. FITC-DEX (150 kDa, Sigma Aldrich) was used at 50 μg/ml, while Anti-Human IgA-FITC (IgG-FITC, Sigma Aldrich) was diluted 1:30 in PBS (37 μg/ml). To assess diffusion through alginate and mixed alginate-mucin hydrogels, straight channels within a PDMS chip were fabricated using a 3D printed mold, where channels were 0.5 mm (height) × 0.5 mm (width) × 10 mm (length) with 2 mm diameter biopsy punched holes (vertical) at both ends. PDMS chips were formed using a Silicone Elastomer Kit (Sylgard) by mixing PDMS elastomer with curing agent at a 9:1 ratio (elastomer:curing agent) and poured into the 3D printed molds (Fig. S3) to be degassed in a vacuum chamber and cured at RT for 48 h. PDMS chips were then incubated in PBS at 37 °C overnight prior to use to remove any air trapped within the chip, and plasma oxidized onto glass slides. Hydrogel mixtures were then formed as mentioned above and injected into the straight channels using a wide bore pipette tip until the hydrogel front reached the end of the channel and incubated at RT for 1 h. Custom pipette tips were 3D printed using an opaque resin with a straight edge and narrowing tip as reservoir holders that were placed at both ends of the straight channels (Fig. S4). Prior to fluorescent imaging, reservoirs at the ends of each channel were filled with half diluted DMEM (in PBS) and incubated at RT for 3 h for the hydrogels to reach the swelling capacity (equilibrate). Fluorescent molecules were diluted to the desired concentrations in half diluted DMEM (in PBS), where the fluorescent mixture was deposited into the end with hydrogel front after removing the half diluted DMEM that was used for hydrogel equilibration using a 25-gauge syringe needle. The diffusion device was then placed into a humidified chamber (single-well plate containing presoaked Kim wipes) and imaged for 6 h at 4-min intervals.

Diffusion profiles (2 mm, starting at the hydrogel-liquid interface) were generated using ImageJ for the first 30 frames (2 h). Fluorescent values were normalized to the fluorescence of a channel only containing fluorescent solution, where a matrix of normalized data was used to calculate the effective diffusion coefficient using a custom MATLAB code (see Supplementary Information) modified from Hettiaratchi et al.^54^ to fit the data to a 1-dimensional diffusion function (Eq. 1), where *erfc* is the complementary error function, F is the fluorescence normalized to the initial timepoint, x is the distance from the reservoir-hydrogel interface (cm), t is elapsed time, and D_eff_ is the effective diffusion coefficient (cm^2^/s).

1 $$F\left( {x,t} \right) \propto erfc\left( {\frac{x}{{2\sqrt {D_{eff} t} }}} \right)$$

### Statistical analysis

All statistical analysis was performed on Graphpad Prism (Version 7.0), where mean values were reported with standard deviation as error bars. Statistical significance for all cell viability tests were determined using multiple t-test. Statistical significance of rheological data and DEX droplet contact angles was determined by performing a one-way and two-way analysis of variance (ANOVA), respectively. A two-way ANOVA was also carried out for effective diffusion coefficient and bacterial abundance data analysis.

## Supplementary Information

Supplementary Information.

## Data Availability

The raw/processed data required to reproduce these findings can be provided upon written request to the corresponding author.
