# Supplementary material for: Characterization of an engineered mucus microenvironment for in vitro modeling of host–microbe interactions
Source: Sci Rep. 2022 Apr 1;12:5515. doi: 10.1038/s41598-022-09198-6 (PMC8975841; doi:10.1038/s41598-022-09198-6)
Supplement: Supplementary file 1 — Supplementary Information. [file 41598_2022_9198_MOESM1_ESM.pdf]

Supplementary

Characterization of an engineered mucus microenvironment for *in vitro* modeling of host–microbe interactions

Andy J. Huang[a], Courtney L. O'Brien[a], Nicholas Dawe[a], Anas Tahir[a], Alison J. Scott[d], Brendan M. Leung[a,b,c]*

[a]School of Biomedical Engineering, Faculties of Medicine and Engineering, Dalhousie University, Halifax, Nova Scotia B3H 4R2, Canada

[b]Department of Applied Oral Sciences, Faculty of Dentistry, Dalhousie University, Halifax, Nova Scotia B3H 4R2, Canada

[c]Department of Pathology, Faculty of Medicine, Dalhousie University, Halifax, Nova Scotia B3H 4R2, Canada

[d]Department of Process Engineering and Applied Science, Faculty of Engineering, Dalhousie University, Halifax, Nova Scotia, B3H 4R2, Canada

***Corresponding Author** Brendan M Leung, Email: bleung@dal.ca

Supplementary material contains 5 pages with 4 figures and MATLAB code.

SUPPLEMENTARY FIGURES

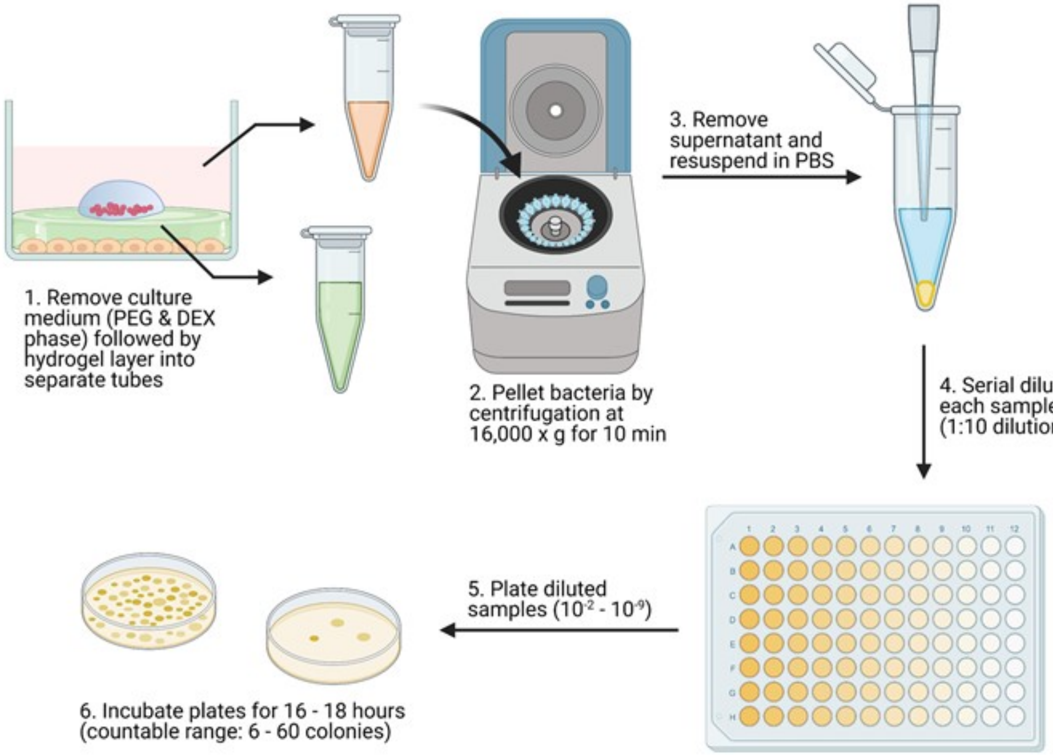

Figure S1. Illustration of techniques used to collect bacteria samples from the culture medium (PEG-phase and DEX-phase) and hydrogel layer of the PEG-DEX ATPS co-culture system for bacteria colony counting to quantify bacterial abundance within each compartment.

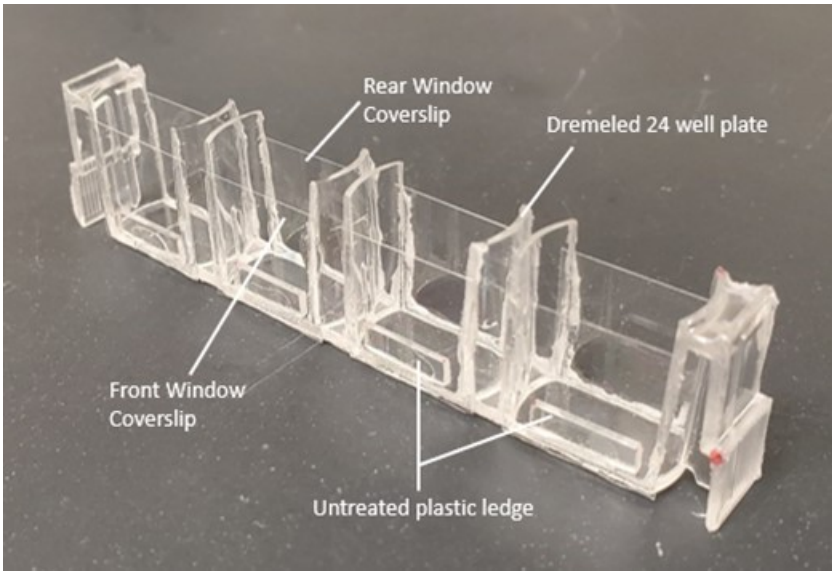

Figure S2. Custom 24-well plate used for sideview imaging of PEG-DEX ATPS on alginate-based hydrogels. Custom plates were cut using a Dremel, where coverslips were epoxied to form a side window with a plastic ledge to prevent meniscus formation after hydrogel deposition.

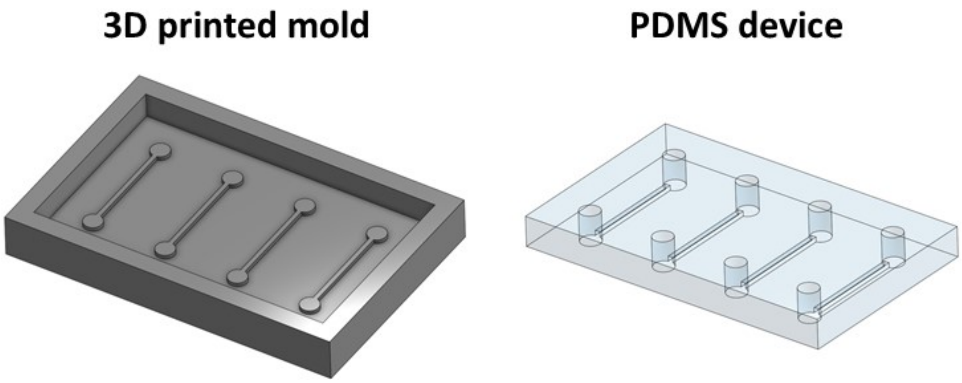

Figure S3. Illustration of the 3D printed mold (left) used to fabricate the straight channel PDMS devices (right) used for diffusivity experiments.

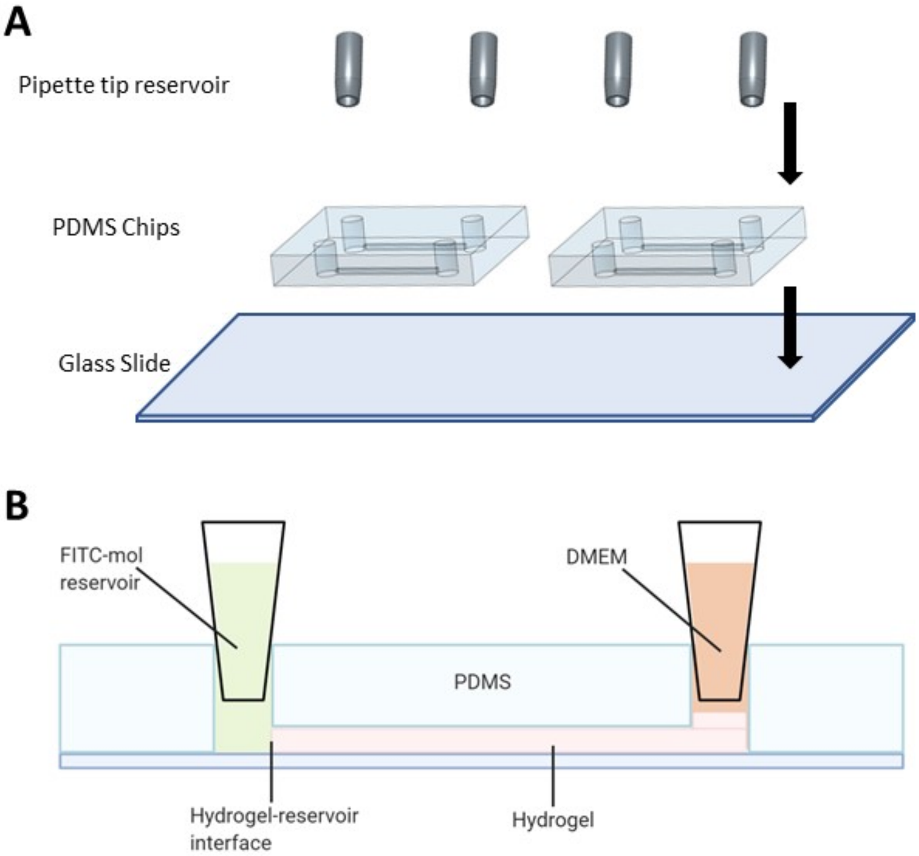

Figure S4. Schematic of PDMS diffusion device set-up. **(A)** PDMS straight channel chip assembly, where PDMS chips were covalently attached to a glass slide, followed by insertion of 3D-printed pipette tip reservoirs. **(B)** Side view of PDMS straight channel containing hydrogel and fluorescein tagged molecules reservoir.

Main Code

```
% Specify the bounds for the diffusion coefficient in cm^2/s
lowbound = 10e-15;
upbound = 10e-4;
% Set options for running the fmincon
% Enable plotting of the results
options =
optimset('PlotFcns',{@optimplotfval,@optimplotstepsize});
% options.StepTolerance = 1e-10;
pmin = 3.5*10e-7;

% pmin = 10^-6;
[pminf,Smin,exitflag,output]=fmincon(@Data_ToMinimize,...
    pmin,[],[],[],[],lowbound,upbound,[],options);

% Data_ToMinimize(pmin);
```

Function to Minimize

```
function S = Data_ToMinimize(p)
load('fluorescence_1')
% distance = distance(2:end);
% matrix = matrix(:,2:end);

% matrix = matrix([1 2 15 30],:);
% time = time([1 2 15 30]);

distance(1,1) = eps;
for i = 1:length(time)
    for j = 1:length(distance)
        if matrix(i,j) == 0
            matrix(i,j) = eps;
        end
    end
end

% matrix = matrix./100;

% Parameter to solve for: diffusion of protein
D_eff = p; % cm^2/s
% Time in seconds for each image
t = 60*time; % s
% Convert distance to centimeters
x = 10*(distance); %cm
```

```
% Calculate the value to plug into our erfc, defined in Clauss,
et al. 1990
% First, create empty vectors
y = zeros(length(t),length(x)); % Value to plug into the
equation
C = zeros(length(t),length(x)); % Calculated concentration, as
fcn of time and position
% Step through each time point to calculate the input variable,
y
% And the resulting concentration, C
for i = 1:length(t)
y(i,:) = x./(2*sqrt(D_eff.*t(i)));
C(i,:) = erfc(y(i,:));
end
% C = 0.1*C;

% Create plots
subplot(2,1,1)
plot(x*10000,matrix,'LineWidth',2)
xlabel('Distance, µm')
ylabel('Normalized Fluorescence')
ax = gca;
ax.LineWidth = 1;
ax.FontSize = 14;

subplot(2,1,2)
plot(x*10000,C,'LineWidth',2)
xlabel('Distance, µm')
ylabel('Normalized Fluorescence')
ax = gca;
ax.LineWidth = 1;
ax.FontSize = 14;

% Error Function
S = 0;
for i = 1:length(x)
S = S + sum(((C(:,i) - matrix(:,i))./matrix(:,i)).^2);
end

end
```
